# Histological evaluation of the regenerative potential of a novel photocrosslinkable gelatin-treated dentin matrix hydrogel in direct pulp capping: an animal study

**DOI:** 10.1186/s12903-024-03868-9

**Published:** 2024-01-19

**Authors:** Eman M. Sedek, Sally Abdelkader, Amal E. Fahmy, Elbadawy A. Kamoun, Samir R. Nouh, Nesma Mohamed Khalil

**Affiliations:** 1 Dental Biomaterials Department, Faculty of Dentistry, Alexandria University, Alexandria, Egypt; 2 Polymeric Materials Research Department, Advanced Technology and New Materials Research Institute, City of Scientific Research and Technological Applications (SRTA-City), New Borg Al-Arab City 21934, Alexandria, Egypt; 3 Nanotechnology Research Center (NTRC), The British University in Egypt, El-Shreouk City, Cairo Egypt; 4 Surgery Department, Faculty of Veterinary Medicine, Alexandria University, Alexandria, Egypt; 5 Oral Biology Department, Faculty of Dentistry, Alexandria University, Alexandria, Egypt

**Keywords:** Dentin matrix, Dentin regeneration, Gelatin, Hydrogel, Injectable scaffold, Pulp capping

## Abstract

**Background:**

To assess histologically the success of the pulp capping approach performed in traumatically exposed dogs’ teeth using a novel injectable gelatin-treated dentin matrix light cured hydrogel (LCG-TDM) compared with LCG, MTA and TheraCal LC.

**Methods:**

Sixty-four dogs’ teeth were divided into two groups (each including 32 teeth) based on the post-treatment evaluation period: group I: 2 weeks and group II: 8 weeks. Each group was further subdivided according to the pulp capping material into four subgroups (*n* = 8), with subgroup A (light-cured gelatin hydrogel) as the control subgroup, subgroup B (LCG-TDM), subgroup C (TheraCal LC), and subgroup D (MTA). Pulps were mechanically exposed in the middle of the cavity floor and capped with different materials. An assessment of periapical response was performed preoperatively and at 8 weeks. After 2 and 8-week intervals, the dogs were sacrificed, and the teeth were stained with hematoxylin-eosin and graded by using a histologic scoring system. Statistical analysis was performed using the chi-square and Kruskal-Wallis tests (*p* = 0.05).

**Results:**

All subgroups showed mild inflammation with normal pulp tissue at 2 weeks with no significant differences between subgroups (*p* ≤ 0.05), except for the TheraCal LC subgroup, which exhibited moderate inflammation (62.5%). Absence of a complete calcified bridge was reported in all subgroups at 2 weeks, while at 8 weeks, the majority of samples in the LCG-TDM and MTA-Angelus subgroups showed complete dentin bridge formation and absence of inflammatory pulp response with no significant differences between them (*p* ≤ 0.05). However, the formed dentin in the LCG-TDM group was significantly thicker, with layers of ordered odontoblasts identified to create a homogeneous tubular structure and numerous dentinal tubule lines suggesting a favourable trend towards dentin regeneration. TheraCal LC samples revealed a reasonably thick dentin bridge with moderate inflammation (50%) and LCG showed heavily fibrous tissue infiltrates with areas of degenerated pulp with no signs of hard tissue formation.

**Conclusions:**

LCG-TDM, as an extracellular matrix-based material, has the potential to regenerate dentin and preserve pulp vitality, making it a viable natural alternative to silicate-based cements for healing in vivo dentin defects in direct pulp-capping procedures.

**Supplementary Information:**

The online version contains supplementary material available at 10.1186/s12903-024-03868-9.

## Background

Dental pulp tissue is a highly specialized tissue that can be affected by various insults, primarily dental caries, and has the capacity to heal against caries or traumatic stimuli, like other connective tissues [1]. Regeneration of the dentin–pulp complex is of paramount importance to restore tooth vitality. Direct pulp capping (DPC) is one of the vital pulp therapy modalities aimed at maintaining the vitality of the pulp through dentin bridge formation [2, 3]. Numerous materials have been considered for DPC, such as tricalcium silicate-based cements (the mineral trioxide aggregate, TheraCal LC and Biodentine), which became the materials of choice for conservative pulp treatment [4, 5]. Despite their benefits, they have some drawbacks, such as the cytotoxicity of freshly prepared calcium silicate-based synthetic materials owing to their high initial pH [6], a lack of spatial control over biological signals required for the homing and differentiation of dental pulpal stem cells (DPSC), and a long setting time [7, 8]. As a result, the development of an alternate material that addresses these drawbacks is anticipated.

Nowadays, there is great interest in the use of natural materials for the treatment of DPC [9, 10]. Tissue engineering with the triad of dental pulp stem cells, bioactive molecules, and scaffolds [11] may provide a useful alternative natural method for DPC. The strategy focuses on developing scaffold-cell complexes to stimulate dental tissue regeneration [12, 13], among which decellularized extracellular matrix (dECM) materials [14, 15] standing out because of their distinct natural structure, biological induction activity, and biocompatibility. A variety of decellularized techniques are used to generate the dECM-based materials, which are derived from an extracellular matrix [16]. It has the ability to control the behavior of tissue-regenerating cells in addition to giving them a three-dimensional scaffold structure [17]. Hydrogel-based scaffolds are a distinct class of three-dimensional, highly water-filled polymeric networks. In addition to having mechanical characteristics and tunable degradation patterns, they are hydrophilic, biocompatible, and capable of being loaded with various bioactive molecules. Additionally, hydrogels are highly elastic and flexible, resembling the extracellular matrix (ECM) of cells, especially the dental pulp [18]. Moreover, to achieve the perfect imitation for dentin regeneration, scaffolds loaded with dentin tissue are the best choice. During the last decade, considerable research has been conducted on TDM, which is a unique kind of decellularized extracellular matrix derived from a dentin matrix. TDM maintains the fundamental architecture of native dentinal tubules, enabling stem cells to attach and offering the required space for the flow of nutrients and metabolic waste [19–22].

Although TDM, as a potential natural material, has certain drawbacks, it is difficult to customize in a pulp-capping procedure. Several studies have attempted to produce formulations of TDM with improved handling properties [23, 24]. In addition, the plasticity was not perfect, making the pulp capping procedure not convenient enough. As a result, delivering TDM in a unique form other than powder-like, such as a paste or hydrogel, may provide the material with better handling properties, allowing it to be in an injectable form without compromising the bioactivity of TDM. Injectability offers an advantage when the defect size is small and irregular because the scaffold could be injected and hardened in the defect area, making it more favorable for clinical application [25]. Furthermore, injectability with a controllable setting time is critical in the DPC technique. Consequently, the fabrication of hydrogels using the photo-crosslinking approach has attracted significant attention within the tissue engineering field [26, 27]. Therefore, this study introduced TDM for the first time as a light-cured hydrogel with gelatin. As recently, gelatin has been used in conjunction with human DPSCs for bone tissue regeneration [28], dentin-pulp complex tissue engineering [11, 29], dentin regeneration [30], and Dental Follicle Stem Cells for tooth root regeneration [31]. Gelatin can support DPSCs adhesion, proliferation, migration, and odontogenic differentiation, as was evident by increased mineralization, ALP activity, and enhanced expression of collagen I (Col I), in addition to being inexpensive [32, 33]. Moreover, gelatin has the ability to maintain its physical characteristics at body temperature due to its thermal stability [34]. Nowadays, gelatin, in conjunction with methacrylate (GelMA), can be polymerized under visible curing light in the presence of a photoinitiator [35]. GelMA is biocompatible, and retains RGD cell-binding motifs and the MMP-binding domain, which support cellular adhesion, proliferation, migration and organization [32, 36, 37].

Gelatin hydrogel with a natural photoinitiating system composed of riboflavin as a photoinitiator under visible light and glycine as a first-time coinitiator with riboflavin loaded with treated dentin matrix powder as a decellularized extracellular matrix is a unique and powerful candidate for dentin pulp complex regeneration because of its similarity to native tissues and astounding biological induction activity. Furthermore, unlike MTA, which is the gold standard for direct pulp capping operations [38], it may be simply injected into the exposed pulp cavity and photopolymerized in situ using ordinary dental curing light, facilitating its clinical translation for dental regeneration. The purpose of the current study was to prepare a novel injectable mixture combining gelatin hydrogel and TDM with a natural photoinitiating system composed of riboflavin and glycine, as no study has introduced TDM as an in situ photocrosslinkable hydrogel with a natural photoinitiating system to be a powerful composite to be used as a pulp-capping agent for dentin regeneration compared with the LCG, MTA, and TheraCal LC.

Based on the study’s results, the null hypothesis of no differences in the regenerative ability of the novel scaffold made of light-cured gelatin loaded with a treated dentin matrix compared with LCG, MTA, and TheraCal LC when used as direct pulp capping materials was rejected as there was a difference in the amount, thickness, and quality of hard tissue formed on exposed pulps with LCG-TDM showing a promising trend to dentin regeneration.

## Materials and methods

### Materials

Gelatin from porcine skin (type A, 300 bloom corresponding to a molecular weight range of 50 to 100 kDa), Glycidyl methacrylate (GC, ≥ 97.0%) and triethylamine were provided by Sigma-Aldrich Chemie GmbH (Steinheim, Germany). Riboflavin and glycine were obtained from Sigma-Aldrich (St. Louis, MO, USA). Dimethyl Sulfoxide (DMSO) was purchased from Fluka Chemie, Germany. Dialysis tubing cellulose membrane (Mwt cut-off 14,000, average diameter 16 mm) was acquired from Merck, Germany. Mineral trioxide aggregate (Angelus, Londrina, PR, Brazil). Theracal LC (Bisco, Schaumburg, IL, USA). Glass ionomer (SDI, Bayswater, Victoria, Australia). Single bond universal adhesive and composite restorative filing (3 M-ESPE, St. Paul, MN, USA). At λmax 460 nm and 1100 mW/cm^2^, a blue light-emitting diode lamp (LED-lamp) (Bluephase, Ivoclar Vivadent, Amhest, NY, USA) was utilized for irradiation. The distance between the irradiation light source and the capped material was almost 0 cm. The irradiation time was ca. ≥ 50 s.

### Methods

#### Sample size calculation

The sample size was calculated assuming 80% study power and 5% alpha error. Holeil et al. [2] reported a mean ± SD dentinal bridge thickness score after 2 months = 1.0 ± 0.00 when treated dentin matrix hydrogel was used and 2.2 ± 0.84 in case of MTA use. Bakhitiar et al. [39] reported dentinal bridge thickness scores when TheraCal was used, which were then used to calculate the mean ± SD score. Based on the comparison of means, the sample size was calculated to be 7 per group and increased to 8 to make up for laboratory processing errors. The total sample size required = number of groups × number per group = 8 × 8 = 64 teeth [40].

#### Fabrication of LCG-TDM hydrogel

**Fig. 1:**
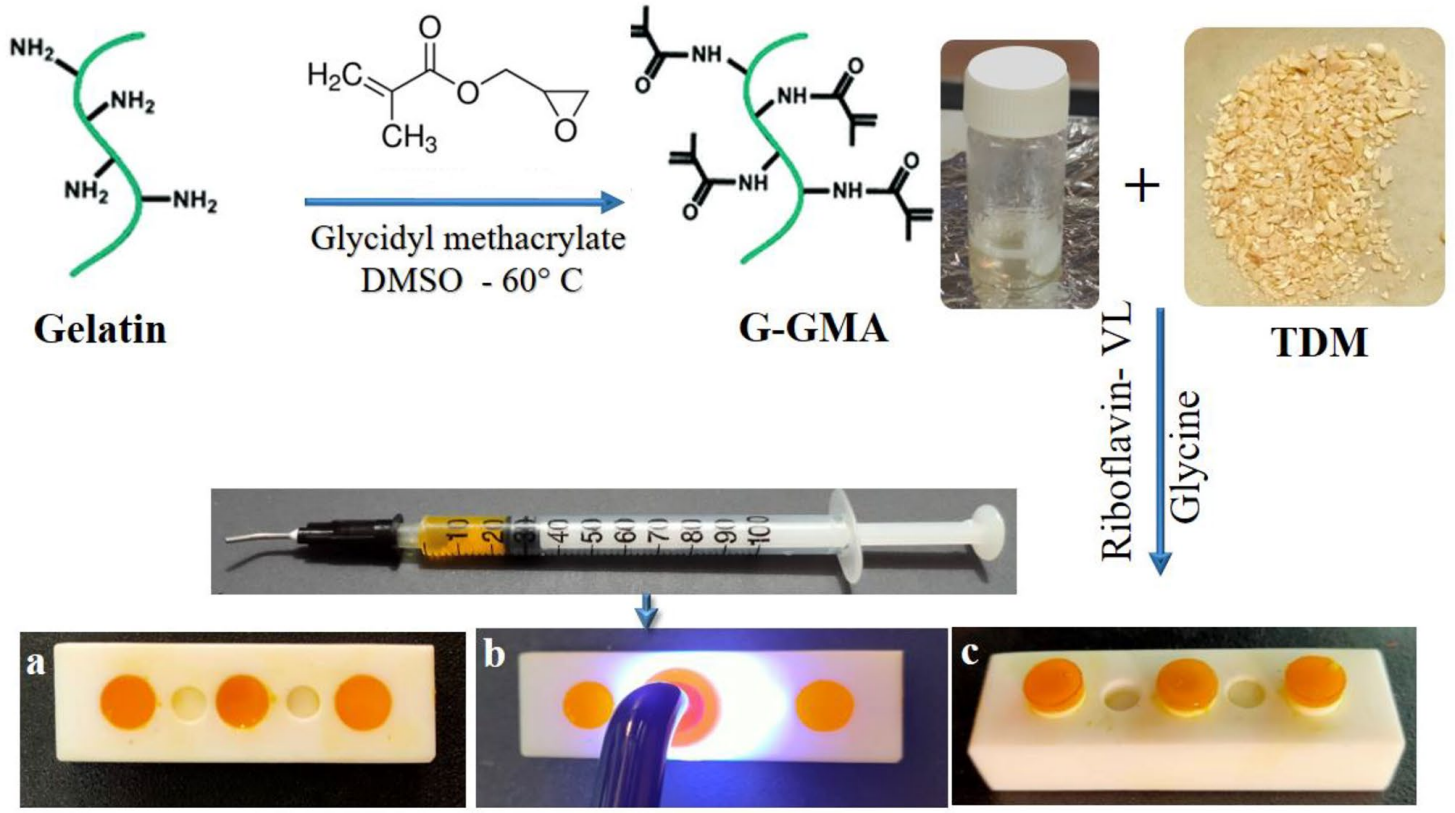
A simplified diagram showing the grafting reaction of gelatin with GMA and the crosslinking of LCG-TDM hydrogel under visible light irradiation

Gelatin (2 g) was dissolved in 150 mL of DMSO under a magnetic stirring (Eneflux-Armtek Magnetics, Inc., USA) speed of 300 rpm at 50 °C for 3 h. After the mixture was fully homogenized, the pH of the mixture was adjusted to 9 with triethylamine. Subsequently, 2 mL (1:0.097 M) of glycidyl methacrylate was mixed into the solution by constant and vigorous stirring at 60 °C for 24 h. Then, the solution was dialyzed for 3 days at 40 °C followed by freeze drying and storage at -20 °C until further use [41–43]. The freeze dried gelatin-glycidyl methacrylate (G-GMA) copolymer powder (15 w/v%) was dissolved in distilled water and then mixed with TDM with a < 76 μm particle size obtained from freshly extracted sound mandibular and maxillary first and second molars from recently discarded 1–2 year-old male dog jaws obtained from the animal house of the Medical Research Institute, Alexandria University, for experimental reasons using a TDM treatment protocol under aseptic conditions as in our previous research [44] at a mass ratio of 1:1. The mixture was optimized with respect to TDM content in order to achieve the maximum quantity of dentinoconductive filler and correspond to the maximum amount of TDM powder that allowed the development of injectable formulations [45]. Then riboflavin (12 µM) as a photoinitiator was added to the mixture as recommended by Hyun H et al. [46] and Kim SH et al. [47]. As one unique characteristic of riboflavin as a photoinitiator is that only small amounts of riboflavin are needed to activate the photocrosslinking process. The mixture was subsequently stirred for 5 min at room temperature in a dark glass bottle to prevent any premature polymerization from the surrounding visible light irradiation [48, 49]. Glycine (0.5 mol%) as a coinitiator was added to the mixture and then stirred until a homogeneous solution was formed. After that, the mixture was injected and photocrosslinked for 60 s using a light curing unit (Fig. 1).

#### Animals

The present study was approved by the Institutional Ethical Committee, Faculty of Dentistry, Alexandria University (IRB No. 00010556-IORG No. 0008839) (0433-April 2022). The authors followed all institutional and international guidelines for animal care and use during this study. The Animal Research: Reporting in Vivo Experiments guidelines (ARRIVE) were also followed up. The maxillary and mandibular left and right permanent premolars, canines and sometimes first molars were used in each quadrant of four adult male mongrel dogs with an approximate weight of 15–20 kg aged 1–2 years for this study. These animals were obtained from the animal house of the Medical Research Institute, Alexandria University. Each dog was subjected to a full physical examination by an expert veterinarian to exclude any diseased dogs and kept under observation in separate cages (1.5 m x 2.5 m x 3 m). They were housed under proper conditions of ventilation, nutrition, cleaning and a 12-hour light/dark cycle, and they were given two meals of soft food daily and clean water. The standard diet regimen was refilled daily throughout the experimental period. The animals were kept in the animal house at the Tissue Engineering Department in the Faculty of Dentistry, Alexandria University, Egypt.

#### Classification of samples, randomization and allocation concealment

**Fig. 2:**
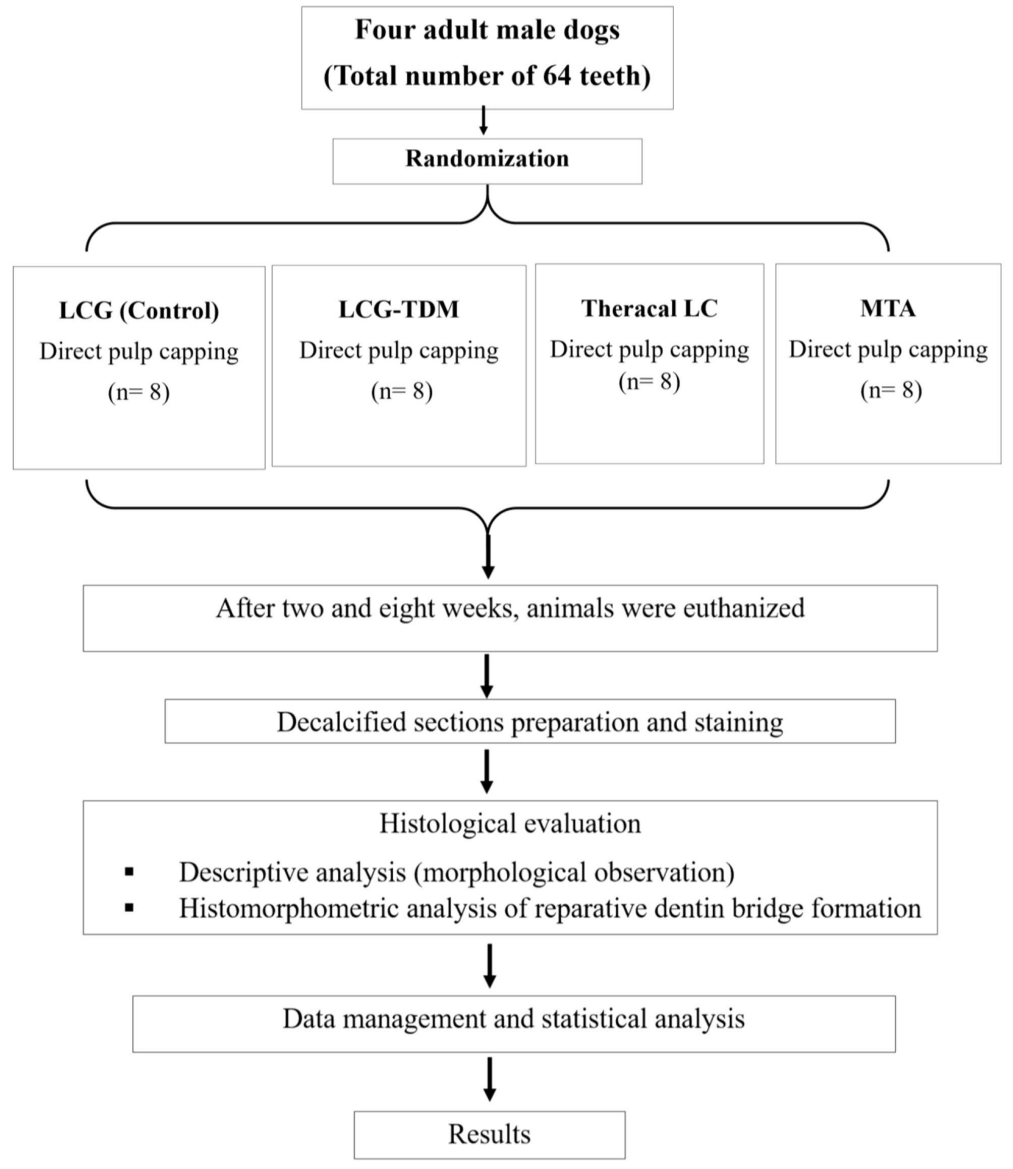
The flow chart of the animal study

The samples (64 teeth) were divided according to the post-treatment evaluation period into two groups (32 teeth each), group I: 2 weeks and group II: 8 weeks. Each group was further subdivided according to the pulp capping material into four subgroups (8 teeth each), with subgroup A (light-cured gelatin hydrogel) as the control subgroup, subgroup B (LCG-TDM), subgroup C (TheraCal LC), and subgroup D (MTA) (Fig. 2). The site of pulp capping was the cervical third of the buccal surface of the selected teeth. Dogs were randomly assigned, using computer generated random numbers to one of the four groups according to the type of direct pulp capping material that was applied.

#### Direct pulp capping procedure

**Fig. 3:**
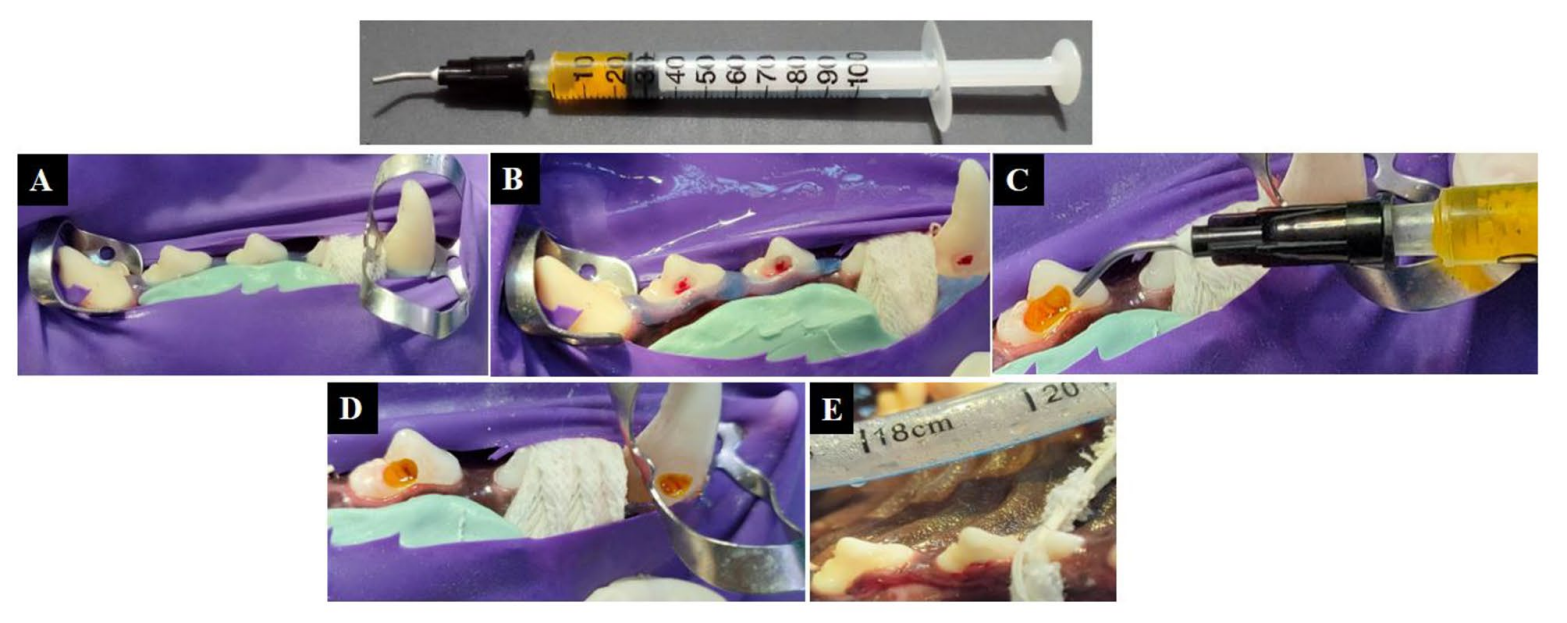
Clinical photographs of the direct pulp capping procedure using LCG-TDM; (**A**) Rubber dam isolation; (**B**) Pulp exposure and vitality confirmation; (**C**) LCG-TDM hydrogel placement; (**D**) LCG-TDM hydrogel after light-cured and removing excess material; (**E**) After final restoration

General anaesthesia was induced by S/C injection of 0.05 mg/kg Atropine sulphate (Atropine sulphate 1%R; ADWIA, Egypt), IM injection of 1 mg/kg Xylazine HCl (Xylaject 2%R; ADWIA, Egypt), and IM injection of 10 mg/ kg Ketamine HCl (KeiranR; EIMC Pharmaceuticals Co., Egypt). The anaesthesia was maintained with incremental doses of Thiopental sodium 2.5% solution (Thiopental sodium R; EIPICO, Egypt) at a dose of 25 mg/kg given intravenously. Before starting the operative protocol, every tooth was radiographically examined for periapical tissues (Digital X-Ray Machine RAY-221, USA). Then, the operative field was covered with a rubber dam and cleaned with 2% chlorhexidine-gluconate (JK Dental, A.R.E.) (Fig. 3A). Class V cavities were prepared on the cervical third of the buccal surface, 0.5-1 mm above the gingival margin, parallel to the cemento-enamel junction with a 2.5 mm width, 3 mm length and 1.5–2 mm depth using a sterile round carbide bur (Komet, Lemgo, Germany). Cavities were deepened, and the pulp tissue was exposed in a standardized manner in the middle of the cavity floor using a sterile, sharp endodontic explorer (DG16, Dental USA Inc., McHenry, IL, USA). To avoid any thermal damage during the cavity preparation process, the tooth and the cutting instruments were heavily irrigated with sterile saline solution. After pulp exposure and vitality confirmation (Fig. 3B), in order to remove any debris, sterile saline was used to rinse the exposure and its surroundings. For total hemostasis, a cotton pellet soaked with NaOCl (JK Dental, A.R.E.) solution was placed over the exposure for one to two minutes [3, 50].

LCG hydrogel without TDM as the positive control group was the pulp capping material employed for subgroups IA and IIA. It was injected over the exposed pulp in one increment (0.5-1 mm) by using an injectable syringe [51, 52]. For subgroups IB and IIB, LCG-TDM hydrogel was used as a pulp capping agent (Fig. 3C and D). In subgroups IC and IIC, the material applied for capping was TheraCal LC and applied following the manufacturer’s instructions. In subgroups ID and IID, MTA-Angelus was used as a pulp capping agent. It was mixed and applied in accordance with the manufacturer’s guidelines. Then, all cavities in the prepared teeth were lined with a thin layer of resin modified glass ionomer cement, then selectively etched with 37% phosphoric acid gel, rinsed, and dried. A single bond universal adhesive was applied and rubbed for 20 s, air-thinned, and light-cured. The resin composite restoration was placed in incremental layers and light-cured (Fig. 3E). All procedures were performed by one experienced operator. Moreover, periapical radiographs were taken at 8 weeks before sacrifice to evaluate the status of the periapical tissues.

#### Histological examination

Animals were sacrificed after 2 and 8 weeks by overdose of a general anaesthetic solution given rapidly by IV route (20 mL of 5% Thiopental sodium). For two weeks, the teeth and the tissues around them were fixed in a 10% buffered formalin solution. After two weeks of fixation, the teeth were decalcified by 17% EDTA solution (New Pharmchemical Co., Egypt) (pH 7.4) for 120 days. Five micron thick serial sections in the linguobuccal plane of the paraffin embedded teeth were stained with hematoxylin-eosin [53, 54]. In order to avoid any potential bias, two blinded expert examiners investigated all the specimens by using coded samples throughout the study. Inter- and intra-examiner reliability was verified using Kappa statistics (Cohen kappa ≥ 0.86). The amount of hard tissue formation at the capping material interface (continuity and thickness), the inflammatory cell response, and the pulp tissue organization were assessed using a light microscope (BX41; Olympus, Tokyo, Japan) that was connected to a high resolution video camera. These evaluations were made using modified criteria based on those of Medina et al. [55] and Nowicka et al. [56]. For each specimen, three serial sections were used for histological and histomorphometric analyses, as a mean was obtained and analyzed at × 40 magnification. The calcified tissue bridge thickness was measured using image analysis software ‘Image-J software’ in the cervical-occlusal direction by the average (in mm) of three linear measurements, one made in the central region of the pulp exposure site and the other two at equal distance from this first measurement [18]. Histomorphological sections were scored from 1 to 4, with 1 being the most desired result and 4 being the least desired result as shown in Table 1.

**Table 1:** Scores and criteria for histological evaluation

Scores	Characterization
	**Morphology and continuity of dentin bridge**
1	Intense hard tissue deposition beneath the exposed area appearing as a complete dentin bridge
2	Formation of discontinuous incomplete dentin bridge extending to more than one-half of the exposure site but not completely closing the exposure site
3	Initial dentin bridge formation extending to not more than one-half of the exposure site
4	No signs of dentin formation
	**Thickness of dentinal bridge**
1	= > 0.25 mm
2	= 0.1–0.25 mm
3	= < 0.1 mm
4	= Partial or absent bridge.
	**Inflammatory cell response**
1	Absent
2	Mild, inflammatory cells only next to pulp exposure site
3	Moderate, inflammatory cells observed in part of coronal pulp
4	Severe, all coronal pulp is infiltrated
	**Pulp tissue organization**
1	Normal or almost normal tissue morphology
2	Disorganization of pulp beneath the cavity
3	Total disorganization of pulp tissue morphology
4	Pulp necrosis

### Statistical analysis

Data were fed to the computer and analyzed through the IBM SPSS software package, version 20.0. (Armonk, NY: IBM Corp). Significance was accepted at *p* = 0.05. Categorical data were represented as numbers and percentages. Chi-square test was applied to compare between two groups. Alternatively, Fisher Exact or Monte Carlo correction test was applied when more than 20% of the cells have expected count less than 5 and Kruskal-Wallis test for abnormally distributed quantitative variables, to compare between more than two studied groups, and post hoc (Dunn’s multiple comparisons test) for pairwise comparisons.

## Results

**Fig. 4:**
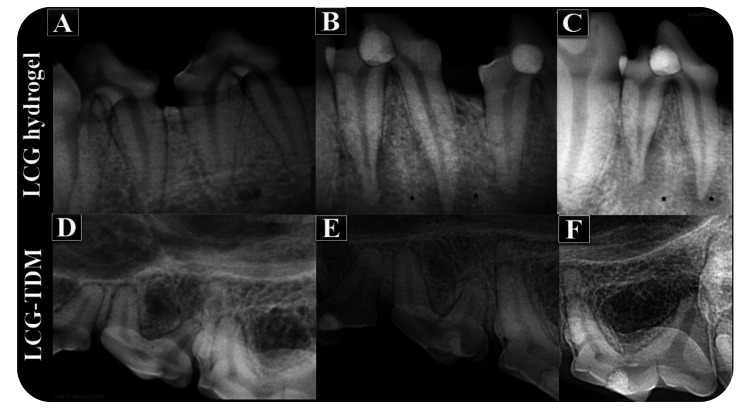
Periapical radiographic examination of teeth treated with LCG and LCG-TDM, respectively. (**A** and **D**) Preoperative periapical radiographs showing sound teeth and normal periapical tissues. (**B** and **C**) Postoperative periapical radiographs showing periapical pathology with root resorption after 8 weeks of treatment with LCG before extraction. (**E** and **F**) Postoperative periapical radiographs showing no sign of periapical pathology after 8 weeks of treatment with LCG-TDM before extraction

**Fig. 5:**
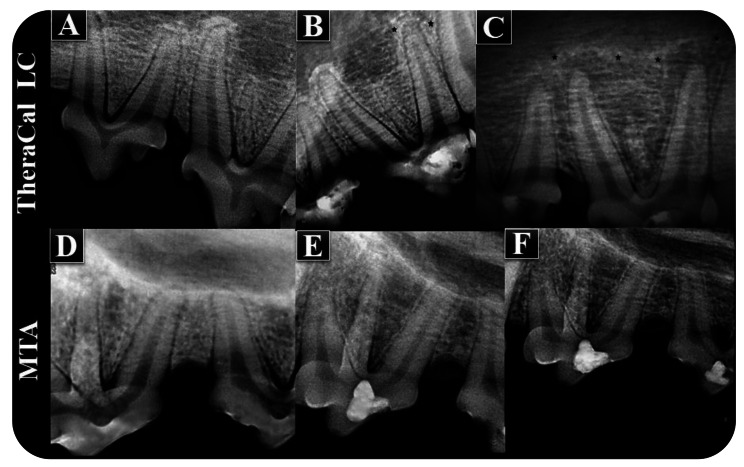
Periapical radiographic examination of teeth treated with TheraCal LC and MTA, respectively. (**A** and **D**) Preoperative periapical radiographs showing sound teeth and normal periapical tissues. (**B** and **C**) Postoperative periapical radiographs showing periapical pathology after 8 weeks of treatment with TheraCal LC before extraction. (**E** and **F**) Postoperative periapical radiographs showing no sign of periapical pathology after 8 weeks of treatment with MTA before extraction

The maxillary and mandibular left and right permanent premolars (total number = 46), canines (total number = 16), and sometimes first molars (total number = 2) were used in each quadrant of four adult male mongrel dogs with an approximate weight of 15–20 kg aged 1–2 years for this study. Prior to sacrifice, the animals were reweighted to ensure they had an approximate normal initial weight, and periapical radiographs were performed on all teeth (8 weeks). Periapical radiographs did not reveal any signs of periapical pathology before the extraction, as shown in Figs. 4 and 5, except for the TheraCal LC and LCG (control) treatment modalities. There was a periapical lesion in the TheraCal LC subgroup and a periapical lesion with root resorption in the LCG subgroup.

### Histological evaluation

The outcome of the histological assessment is explained regarding the inflammatory reaction, pulp tissue organization, hard tissue formation, and thickness of the dentinal bridge. Data were collected, tabulated, statistically analyzed and represented in Table 2.

**Fig. 6:**
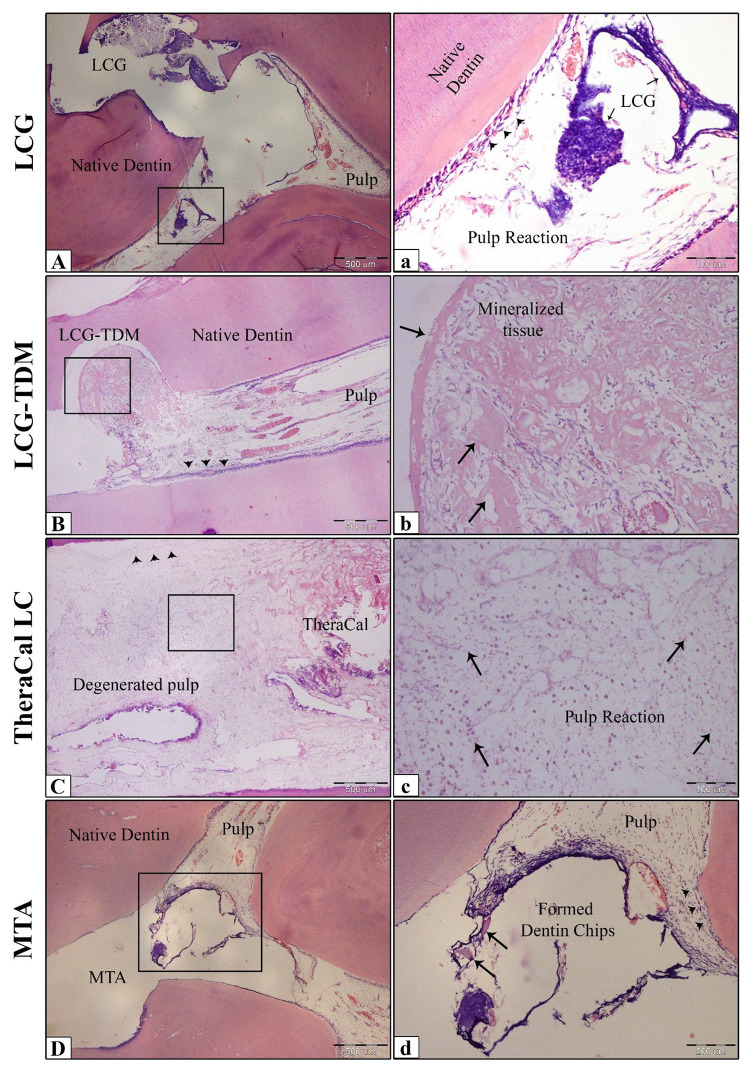
Photomicrographs of dog pulp capped with LCG (**A& a**), LCG-TDM (**B& b**), TheraCal LC (**C& c**), and MTA (**D& d**) at 2-week interval. LCG showing normal pulp structure with a well-organized odontoblastic layer (arrowheads) and no signs of newly formed tissue adjacent to the pulp-exposed area, while LCG-TDM showing initial dentin bridge formation next to the pulp-exposed area with scattered calcified fragments (arrows). TheraCal LC exhibiting degenerated pulp with loss of normal pulp organization (arrows) and disturbed odontoblastic layer (arrowheads), MTA displaying normal pulp structure with a regular odontoblastic layer (arrowheads) and newly formed dentin chips (arrows). H&E: (A, B, C, D ×40) and (a, b, c, d ×200)

After 2 weeks, normal pulp structure persisted in almost all of the teeth in all subgroups except for the TheraCal LC subgroup, which showed less organized pulp tissues with focal necrotic areas as well as no evidence of a complete or even partial calcified bridge adjacent to the pulp-exposed area in the TheraCal LC and LCG subgroups. Mineralized tissue was found in the LCG-TDM subgroup in the form of an initial dentin bridge next to the pulp-exposed area, in addition to scattered calcified fragments surrounded by pulp tissue. MTA revealed newly generated dentin chips that were dispersed towards the pulp-exposed site. A regular odontoblastic layer was detected in the LCG, LCG-TDM, and MTA subgroups. In almost all sections, the adjacent pulp tissues superficial and deeper parts either had no or a mild inflammatory cell infiltrate in the deeper layers of the pulp, where polymorph-nuclear leukocytes were observed with some dilated blood vessels (Fig. 6), except for the TheraCal LC subgroup, which showed mild to moderate inflammatory cell infiltrate. There was no significant difference between all pulp capping materials in this time interval regarding the inflammatory cell response and pulp tissue organization, except for the TheraCal LC subgroup.

**Table 2:** Comparison between the studied groups according to different parameters in the two studied periods

	LCG		LCG-TDM		TheraCal		MTA		*P* value	
	2 weeks (*n* = 8)	2 months (*n* = 8)	2 weeks (*n* = 8)	2 months (*n* = 8)	2 weeks (*n* = 8)	2 months (*n* = 8)	2 weeks (*n* = 8)	2 months (*n* = 8)	2 weeks (*n* = 8)	2 months (*n* = 8)
**Reparative tissue formation**	4.0 ± 0.0	4.0^a^ ± 0.0	3.3^b^ ± 0.5	1.0^b^ ± 0.0	4.0 ± 0.0	2.4^a^ ± 0.5	3.5^b^ ± 0.5	1.1^b^ ± 0.4	^H^*p* = 0.002^*^	^H^*p*<0.001^*^
Score 1	0 (0.0%)	0 (0.0%)	0 (0.0%)	8 (100.0%)	0 (0.0%)	0 (0.0%)	0 (0.0%)	7 (87.5%)	^MC^*p* = 0.001^*^	^MC^*p*<0.001^*^
Score 2	0 (0.0%)	0 (0.0%)	0 (0.0%)	0 (0.0%)	0 (0.0%)	5 (62.5%)	0 (0.0%)	1 (12.5%)		
Score 3	0 (0.0%)	0 (0.0%)	6 (75.0%)	0 (0.0%)	0 (0.0%)	3 (37.5%)	4 (50.0%)	0 (0.0%)		
Score 4	8 (100.0%)	8 (100.0%)	2 (25.0%)	0 (0.0%)	8 (100.0%)	0 (0.0%)	4 (50.0%)	0 (0.0%)		
***p***_**0**_	–		^MC^*p*_0_=0.001^*^		^MC^*p*_0_<0.001^*^		^MC^*p*_0_<0.001^*^			
**Thickness of dentinal bridge**	4.0 ± 0.0	4.0^a^ ± 0.0	3.3^b^ ± 0.5	1.0^c^ ± 0.0	4.0 ± 0.0	2.6^b^ ± 0.5	3.5^b^ ± 0.5	1.9^bc^ ± 0.0	^H^*p* = 0.002^*^	^H^*p*<0.001^*^
Score 1	0 (0.0%)	0 (0.0%)	0 (0.0%)	8 (100.0%)	0 (0.0%)	0 (0.0%)	0 (0.0%)	1 (12.5%)	^MC^*p* = 0.001^*^	^MC^*p*<0.001^*^
Score 2	0 (0.0%)	0 (0.0%)	0 (0.0%)	0 (0.0%)	0 (0.0%)	3 (37.5%)	0 (0.0%)	7 (87.5%)		
Score 3	0 (0.0%)	0 (0.0%)	6 (75.0%)	0 (0.0%)	0 (0.0%)	5 (62.5%)	4 (50.0%)	0 (0.0%)		
Score 4	8 (100.0%)	8 (100.0%)	2 (25.0%)	0 (0.0%)	8 (100.0%)	0 (0.0%)	4 (50.0%)	0 (0.0%)		
***p***_**0**_	–		^MC^*p*_0_=0.001^*^		^MC^*p*_*0*_<0.001^*^		^MC^*p*_0_<0.001^*^			
**Inflammatory cell response**	1.3^b^ ± 0.5	4.0^a^ ± 0.0	1.1^b^ ± 0.4	1.0^c^ ± 0.0	2.5^a^ ± 0.5	2.4^b^ ± 0.5	1.3^b^ ± 0.5	1.0^c^ ± 0.0	^H^*p* = 0.002^*^	^H^*p*<0.001^*^
Score 1	6 (75.0%)	0 (0.0%)	7 (87.5%)	8 (100.0%)	1 (12.5%)	1 (12.5%)	6 (75.0%)	8 (100.0%)	^MC^*p* = 0.003^*^	^MC^*p*<0.001^*^
Score 2	2 (25.0%)	0 (0.0%)	1 (12.5%)	0 (0.0%)	2 (25.0%)	3 (37.5%)	2 (25.0%)	0 (0.0%)		
Score 3	0 (0.0%)	0 (0.0%)	0 (0.0%)	0 (0.0%)	5 (62.5%)	4 (50.0%)	0 (0.0%)	0 (0.0%)		
Score 4	0 (0.0%)	8 (100.0%)	0 (0.0%)	0 (0.0%)	0 (0.0%)	0 (0.0%)	0 (0.0%)	0 (0.0%)		
***p***_**0**_	^MC^*p*_0_<0.001^*^		^FE^*p*_0_=1.000		^MC^*p*_0_=1.000		^FE^*p*_0_=0.467			
**Pulp tissue organization**	1.3^b^ ± 0.5	4.0^a^ ± 0.0	1.1^b^ ± 0.4	1.0^c^ ± 0.0	2.4^a^ ± 0.7	1.9^b^ ± 0.6	1.1^b^ ± 0.4	1.1^bc^ ± 0.4	^H^*p* = 0.001^*^	^H^*p*<0.001^*^
Score 1	6 (75.0%)	0 (0.0%)	7 (87.5%)	8 (100.0%)	1 (12.5%)	2 (25.0%)	7 (87.5%)	7 (87.5%)	^MC^*p* = 0.004^*^	^MC^*p*<0.001^*^
Score 2	2 (25.0%)	0 (0.0%)	1 (12.5%)	0 (0.0%)	3 (37.5%)	5 (62.5%)	1 (12.5%)	1 (12.5%)		
Score 3	0 (0.0%)	0 (0.0%)	0 (0.0%)	0 (0.0%)	4 (50.0%)	1 (12.5%)	0 (0.0%)	0 (0.0%)		
Score 4	0 (0.0%)	8 (100.0%)	0 (0.0%)	0 (0.0%)	0 (0.0%)	0 (0.0%)	0 (0.0%)	0 (0.0%)		
***p***_**0**_	^MC^*p*_0_<0.001^*^		^FE^*p*_0_=1.000		^MC^*p*_0_=0.409		^FE^*p*_0_=1.000			

^MC^*p*, *p* value for Monte Carlo for comparison between the different studied groups

^MC^*p*_0_, *p* value for Monte Carlo for comparison between 2 weeks and 2 months

^FE^*p*_0_, *p* value for Fisher Exact for comparison between 2 weeks and 2 months

^H^*p*, *p* value for Kruskal-Wallis test, pairwise comparison between every 2 groups was done using Post hoc test (Dunn’s for multiple comparisons test)

Means with common letters are not significant (i.e., means with different letters are significant).

*Statistically significant at *p* ≤ 0.05

**Fig. 7:**
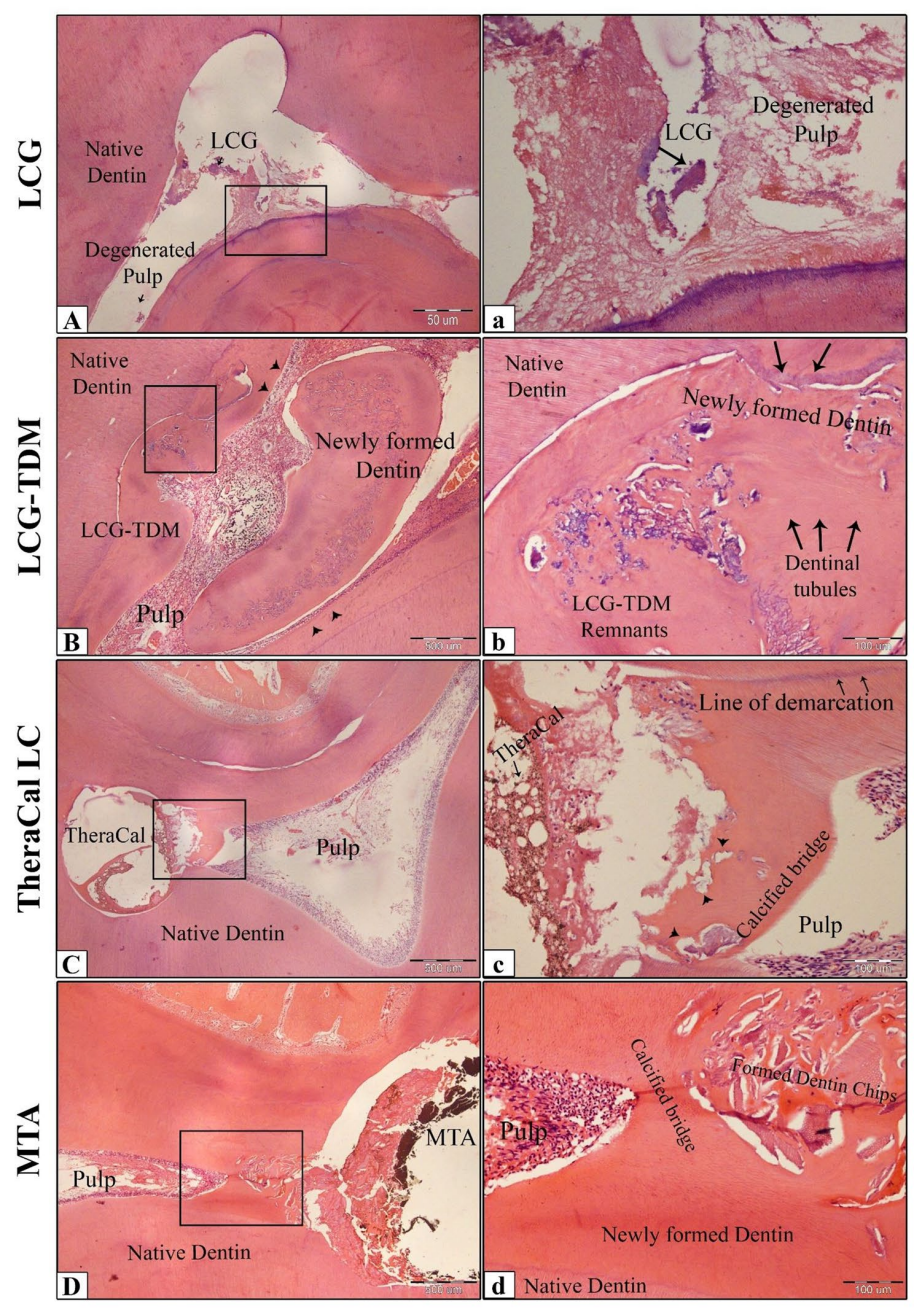
Photomicrographs of dog pulp capped with LCG (**A& a**), LCG-TDM (**B& b**), TheraCal LC (**C& c**), and MTA (**D& d**) at 8-week interval. (**A**) Showing pulp degeneration with remnants of LCG. (**B**) Showing intense complete dentin bridge formation with well-organized odontoblasts (arrowheads) and numerous dentinal tubule lines (below arrows). (**C**) Exhibiting discontinuous dentin bridge (arrowheads) and disorganization of pulp tissue beneath the cavity. (**D**) Showing complete dentin bridge formation. Note the hard tissue bridge incorporating dentin chips. H&E: (A, B, C, D ×40) and (a, b, c, d ×200)

After 8 weeks, the adjacent pulp tissue was mostly normal and had no inflammatory cells among its structural components in the LCG-TDM and MTA subgroups (Fig. 7B, D). However, it was observed the disorganization of pulp tissue beneath the cavity in the TheraCAL LC subgroup (62.5%) with moderate inflammatory cells detectable in part of coronal pulp. The LCG subgroup had degenerated pulp tissue, with almost all sections exhibiting fibrosis and necrosis with a destructive odontoblastic layer (Fig. 7A); they also showed severe inflammation localized directly below the exposure site and scattered in the remaining pulp tissues. Additionally, no calcified tissues were formed in the LCG subgroup. An intense hard tissue was formed in all teeth in the LCG-TDM and MTA subgroups, with no significant difference between them (*P* ≤ 0.05), but with differences in thickness and quality (Table 2). The thickness of hard tissue formed subjacent to LCG-TDM was greater than that of MTA subgroups with a homogenous tubular structure with numerous dentinal tubule lines that are visible in the formed dentin, and odontoblasts appeared to be arranged in a palisaded pattern with columnar cell bodies at the dentin-pulp interface (Fig. 7B). Moreover, there is an area showing tubular structures (Fig. 7b: above arrows) having organized vertically arranged parallel tubules mimicking a tubular-like dentine matrix. TheraCAL LC subgroup exhibited a reasonably thick structure of approximately < 0.1 mm in 62.5% of the samples, extending to more than one-half of the exposure site but not completely closing the exposure site in most of the teeth (62.5%), with scattered globules of newly formed calcified deposit (Fig. 7C).

## Discussion

In some clinical circumstances, vital pulp therapy involves directly applying a biomaterial to the exposed pulp site (direct pulp capping), with the ultimate goal of preserving pulp vitality by protecting the dentin-pulp complex [57]. Histological examination is the gold standard for evaluating the pulp reaction to the test material; as a result, pulp response must be addressed in vivo investigations when novel materials are used for direct pulp capping. Several researchers believe that clinical parameters alone are insufficient to predict the long-term outcome of pulp-capping treatments. Clinically, it is difficult to identify the teeth in which inflammation complicates healing. As a result, the only way to make a vital assessment of the impact of the pulp-capping materials is through histological analysis [58, 59]. This study was conducted on dogs since their teeth composition and growth are similar to that of humans, with large-sized teeth, good accessibility, and a large number of teeth, that minimize the number of animals used in the study [60, 61].

The current histological study used intact teeth in healthy dogs under controlled conditions to avoid the interference by any confounding factors and followed a strict protocol for the direct pulp-capping procedure that included rubber dam isolation, disinfection of the operative field with 2% chlorhexidine-gluconate, and standardized pulp exposure size, as have been carried out in previous studies. During this study, two evaluation periods were selected; the first (2 weeks) was selected to assess pulp tissue response in terms of the material’s biocompatibility, the growth of new odontoblasts, the differentiation process, and the newly formed calcified deposit. The remaining period (8 weeks) was used to evaluate the establishment of the hard tissue barrier [2, 3, 62].

At 2 weeks, the samples of the subgroups (LCG, LCG-TDM, and MTA) demonstrated mild to no inflammatory cell infiltration with appropriate pulp tissue organization. These findings could be attributed to the natural composition of the constructed scaffolds (LCG and LCG-TDM), as gelatin is an insipid protein derived from collagen. It has been proven that gelatin-based hydrogels preserve their structure as basic polymer chains that can be mixed to enable biocompatibility, low immunogenicity, rapid biodegradation, and influencing cell spreading, proliferation, release, recruitment, and viability, allowing their use in tissue engineering [63]. Moreover, gelatin methacrylate-based scaffold has been proposed as a biocompatible material for use in regenerative endodontics [64–66]. This is in accordance with Monterio et al. [67], who found that higher-stiffness GelMA hydrogels seeded with odontoblast-list cells (OD21) are shown to have higher cell spreading, proliferation, and viability close to dentinal walls. Furthermore, light-cured gelatin and light-cured gelatin with TDM scaffolds are constructed from a natural photoinitiating system based on riboflavin and glycine. The photoinitiator riboflavin is a naturally occurring yellow pigment found in numerous plants and microorganisms [47]. Because of its biocompatibility and water solubility, it has been widely used in the biomedical field. Consequently, using riboflavin as a photoinitiator to create hydrogels would be completely biocompatible and even beneficial. Additionally, as a coinitiator, glycine is the most fundamental amino acid and an essential component of critical biological molecules, in addition to being a key element of many metabolic reactions [68]. Therefore, after two weeks, the LCG and LCG-TDM subgroups exhibited favorable behavior and orderly pulp tissue when coming into contact with pulp tissue. This is in accordance with Yang et al. [64], who used GelMA to fabricate hydrogel microspheres by the electrostatic microdroplet method to be applied in endodontic regeneration. In the microspheres, the encapsulated hDPSCs could effectively adhere, spread, proliferate, and secrete extracellular matrix proteins. Regarding the MTA subgroup, calcium silicate cements release a high amount of calcium hydroxide during setting, which is an irritant to pulp tissues. This might explain the mild inflammation that occurred during the early evaluation period [2, 69]. Similar findings were previously reported by Abo El-Mal et al. [3], who claimed that exposed pulp during the initial setting of calcium silicate cements experiences a slight chemical injury due to hydroxyl ions from released calcium hydroxide. TheraCal LC subgroup exhibited mild to moderate inflammation after two weeks with a disturbed odontoblastic layer. These findings mean that TheraCal LC material is less biocompatible than LCG, LCG-TDM, and MTA-Angelus. This may be due to the presence of resin (acrylic monomer Bis-GMA) in its composition [54, 70]. These results are in agreement with Lee et al. [70], who evaluated and compared pulpal responses to ProRoot MTA, RetroMTA, and TheraCal in dog partial pulpotomy models. The results revealed that TheraCal specimens had extensive inflammation and less favorable odontoblastic layer formation.

In respect to the dentin bridge, the absence of a complete or partial calcified dentin bridge was reported for the LCG and TheraCal LC subgroups. Abo El-Mal et al. [3] also found similar results and stated that either complete or partial dentin bridge was not detected for calcium hydroxide and calcium silicate-based cements at 2 weeks. Both LCG-TDM and MTA subgroups had partly calcified tissue; the LCG-TDM showed thin framework-like mineralized tissue, whereas the MTA had dispersed calcific deposit. This is consistent with the findings of Holiel et al. [2], who observed at 2 weeks mild signs of tissue formation next to the pulp-exposed area that included dense vertical sheets of fine fibrils when using TDM alginate hydrogel, Biodentine, and MTA-Angelus on human premolar teeth. Also, Parirokh et al. [71] and Chacko et al. [72] reported neodentine bridge formation at 2 weeks.

Results at 8 weeks confirmed the favorable outcome of the LCG-TDM and MTA subgroups in terms of the complete absence of inflammation, well-organized pulp tissue, thicker and continuous dentin bridge, and the presence of an odontoblast-like cell layer in almost all of the samples. In the MTA subgroup, once the cements are set, the release of irritating calcium hydroxide decreases over time, providing a more favorable environment for pulp healing and obtaining more organized pulp tissue [3, 69]. Furthermore, the findings of the current study showed that MTA could effectively induce reparative dentin synthesis, reflecting the strong biocompatible and bioactive nature of this cement and its ability to provide an appropriate surface to adhere, recruit, and organize odontoblast-like cells before cell differentiation and new dentine bridge formation [2, 3]. These findings are in agreement with Danesh et al. [73] and Faraco et al. [74] who investigated the response of dental pulp capped with MTA in dog teeth. These studies revealed that at 8 weeks the pulp capped with MTA showed a healing process with complete dentin bridge formation.

However, the thickness and quality of the dentin bridge were significantly higher in the LCG-TDM subgroup at 8 weeks compared to the MTA subgroup. Teeth capping with LCG-TDM showed a positive trend towards dentin regeneration, as one important prognostic feature for dentin regeneration is the abundance of dentinal tubules, as well as cytological features of the odontoblast cells, as reported by da Silva et al. [75] and Iohara et al. [76]. This is in accordance with Holiel et al. [2], who evaluated the regenerative ability of a novel TDM hydrogel as a pulp-capping agent compared with Biodentine and MTA. The histological analysis after 2 months showed complete dentin bridge formation. However, the formed dentin was significantly thicker with the TDM hydrogel group, with layers of well-arranged odontoblasts. This is consistent with the findings of Chen et al. [23], who reported that pulp capping with treated dentin matrix paste resulted in better and thicker dentin bridge regeneration with more uniform structural dentin and free of tunnel defects. These results may be attributed to the greater bioactivity and biocompatibility of LCG-TDM, as well as its non-immunogenicity and abundance of potentially dentinogenetic elements [19, 77]. Previous research confirmed that TDM may release dentinogenesis-related proteins, including biglycan, collagen I (COL-1), dentin sialophosphoprotein (DSPP), dentin matrix protein-1 (DMP1), transforming growth factor-β 1 (TGF-β1), and decorin (DCN). These dentinogenesis-related factors are not only crucial for the growth and differentiation of DPSCs into odontoblasts but also create a three-dimensional network that forms new dentin and controls mineralization throughout dentin formation and regeneration [78]. Furthermore, opened dentinal tubules and a loosened collagen matrix in TDM following demineralization, as shown in previous scanning electron microscopy microphotographs [44], could act as channels for releasing these bioactives, and the exposed dense collagen fibers may act as favorable homing for cell attachment [19]. LCG-TDM revealed a great deal as a powerful bioactive pulp capping material, as focal calcifications were most prominently shown in teeth capped with LCG-TDM when accidental pushing of LCG-TDM inside the pulp tissues during capping procedures caused these traces to act as a nidus for calcifications to occur with well-palisaded odontoblast cells on both sides of these calcifications. As a result, LCG-TDM based scaffold induced a natural biological regeneration for the reconstitution of a normal tissue continuum.

Additionally, at both time periods, the LCG-TDM specimens showed almost no infiltration of inflammatory cells, indicating the material’s early and delayed biocompatibility with denser and better organized pulp tissue. This is attributed to the natural composition of the developed scaffold with a riboflavin/glycine natural photoinitiating system, which also affects LCG-TDM’s biocompatibility.

In spite of the biocompatibility of the LCG hydrogel at two weeks, LCG after 8 weeks revealed severe inflammatory cell infiltration along with almost full pulp degradation and necrosis. In addition, there is an absence of complete or partial hard tissue formation. This could be attributed to the absence of signaling and bioactive TDM molecules that could recruit the formative cells to induce dentin regeneration, which acts as a barrier to protect the underlying pulp and maintain pulp vitality. As a result, LCG without TDM was unable to promote dentin regeneration and consequently failed to preserve pulp vitality in a delayed manner. This comes in agreement with Maria et al. [79], who found that incorporation of fibronectin into the collagen/gelatin hydrogel had potent bioactive and chemotactic effects on dental pulp regeneration. In contrast, Monteiro et al. [80] used GelMA hydrogel in combination with dental pulp stem cells (DPSCs) to enhance the regeneration of osteochondral defects in the rabbit TMJ. The result demonstrated that there was no statistical difference in a regenerative enhancement in TMJ defects regarding the use of GelMA with or without DPSCs, but the experimental group (GelMA/DPSCs) had the greatest mean healing score.

The formed reparative dentin in direct pulp capping procedures serves as a physical and natural barrier protecting the underlying pulp. The dentin bridge in the TheraCal LC subgroup exhibits a reasonably thick structure extending to more than one-half of the exposure site but not completely closing the exposure site. As a result, it was unable to serve as the physical barrier protecting the underlying pulp, which allowed inflammatory cells to infiltrate and pulp tissue to become less organized. Subsequently, TheraCal LC samples at the 8-week delayed response had decreased biocompatibility. Also, the inferior biocompatibility could be associated with the presence of the acrylic monomer Bis-GMA in the material. The presence of resin in the pulp capping agent could persist unpolymerized, resulting in unfavourable pulpal reactions that cause inflammation and pulp toxicity [81]. This is consistent with the findings of Alazrag et al. [54], who reported that TheraCal LC demonstrated score 3 inflammatory cell infiltration with limited biocompatibility with respect to MTA-Angelus and Biodentine after three months. Moreover, only 33% of specimens had a full dentin bridge, according to an assessment of pulpal responses in dog partial pulpotomy cases [70]. It was found that TheraCal LC produced the least desirable pulpal responses compared to MTA. Kunert et al. [81] also found similar results and stated that TheraCal LC specimens often displayed less favourable odontoblastic layer formation, widespread inflammation, and lower quality calcific barrier formation. In addition, these findings coincide with another study showed that TheraCal is toxic to pulp fibroblasts and has a stronger inflammatory effect and a lower bioactive capacity than Biodentine when used as direct pulp capping [82].

Based on the study’s results, the null hypothesis of no differences in the regenerative ability of the novel scaffold made of light-cured gelatin loaded with a treated dentin matrix compared with LCG, MTA, and TheraCal LC when used as direct pulp capping materials was rejected as there was a difference in the amount, thickness, and quality of hard tissue formed on exposed pulps with LCG-TDM showing a promising trend to dentin regeneration. The potential therapeutic significance of these findings is that LCG-TDM hydrogels come on to be a viable extracellular matrix-based scaffolds for stimulating dentin tissue regeneration and preserving pulp vitality in DPC as an in situ photocrosslinkable hydrogel with more favorable applicability and a shorter setting time.

The regenerative potential of TDM has been extensively studied, but more research is still needed to fully understand the underlying mechanisms, specific regeneration processes, and possible clinical applications. It is recommended to explore all levels of mechanisms in more detail, from molecules to tissues, from signaling pathways to function performance, and from the microenvironment to structural construction. Furthermore, the use of TDM as a nanotechnology for antimicrobial purposes is still not investigated for improving endodontics’ future [83].

The main limitation of this study is the shorter evaluation periods (early and late). Therefore, longer follow-up periods (one month and three months) are recommended with direct pulp capping techniques for better evaluation of the regenerative capability of the material. Also, it should be considered that the present research was conducted under optimal conditions, where all teeth used were intact and the capped pulps were healthy when exposed without concern for the preoperative state of the inflamed versus non-inflamed pulp. More information is needed to assess the impact of preoperative pulp inflammation [84]. Therefore, further investigations using longer observation intervals, including carious teeth complicated with pulpal inflammation, are required to confirm the current results.

## Conclusions

As an extracellular matrix-based bio-material, LCG-TDM showed great promise as a bioactive pulp capping material, as a combination between gelatin and TDM with riboflavin and glycine in the form of a light-cured injectable hydrogel, having a unique natural structure, astounding biological induction activity, benign biocompatibility, the capability to better regulate the dentinogenesis process, and achieving efficient dentin regeneration with preservation of pulp vitality. Thus, it might serve as a viable natural alternative material for silicate-based cements in restoring in vivo dentin defects in direct pulp capping procedures in clinical applications.

### Electronic supplementary material

Below is the link to the electronic supplementary material.

Supplementary Material 1

## Data Availability

All data generated or analyzed during this study are included in this published article and its Additional file.

## Abbreviations

TDM: Treated dentin matrix
LCG-TDM: Light-cured gelatin hydrogel loaded with treated dentin matrix
DPC: Direct pulp capping
G: Gelatin
GMA: Glycidyl methacrylate
VL: Visible light
ECM: Extra cellular matrix
MTA: Mineral trioxide aggregate
TheraCal LC: Calcium Silicate Light cure
HE: Hematoxylin-eosin
SD: Standard deviation
GelMA: Gelatin methacrylate
RGD: Arginylglycylaspartic acid
MMP: Matrix metalloproteinase
ALP: Alkaline phosphatase
